# Metabolic syndrome and risk of incident dementia in EPIC‐Norfolk: the role of age and exposure duration

**DOI:** 10.1002/alz.094616

**Published:** 2025-01-09

**Authors:** Danial Qureshi, Robert Luben, Shabina Hayat, Robert Talarico, Naomi Allen, Elzbieta Kuzma, Thomas J Littlejohns

**Affiliations:** ^1^ University of Oxford, Oxford, England United Kingdom; ^2^ University of Cambridge, Cambridge United Kingdom; ^3^ University College London, London, England United Kingdom; ^4^ Institute for Clinical Evaluative Sciences (ICES), Ottawa, ON Canada; ^5^ University of Oxford, Oxford United Kingdom; ^6^ University Medical Center Hamburg‐Eppendorf, Hamburg Germany; ^7^ University of Hamburg, Hamburg Germany

## Abstract

**Background:**

Metabolic syndrome (MetS) might be a modifiable risk factor for dementia. However, the impact of mid‐life versus late‐life MetS and the duration living with MetS on dementia risk remains underexplored. This study investigated whether the association between MetS and risk of dementia differs across mid‐life versus late‐life, and to explore how duration of MetS influences this risk.

**Method:**

We conducted a prospective study using data from the European Prospective Investigation into Cancer—Norfolk (EPIC‐Norfolk). In full cohort analyses, we studied 20,150 dementia‐free adults aged ≥50 years who attended baseline assessments. Group‐based trajectory analysis was performed on 12,756 participants who attended ≥2 health assessments over 20 years. MetS was defined as the presence of ≥3 of the following: elevated waist‐circumference, triglycerides, blood‐pressure, HbA1c, or reduced HDL‐cholesterol. Incident all‐cause dementia was ascertained using hospital inpatient, death, and mental healthcare records.

**Result:**

The mean (standard deviation [SD]) age of participants was 62.6 (7.5) years, and 54% were female. Over 25 years of follow‐up (mean 18.8 [SD 6.3]), 2,653 participants developed dementia. In the overall cohort (all ages: 50‐79 years), MetS was associated with an increased risk of dementia (Hazard‐Ratio [HR], 1.11, 95% Confidence‐Interval [CI]: 1.01‐1.21). Age‐specific analyses revealed that this risk only remained in those with late mid‐life MetS (age 60‐69: HR, 1.21, 95%CI: 1.05‐1.39) but not in early mid‐life (age 50‐59: HR, 1.12, 95%CI: 0.87‐1.43) or late‐life (age 70‐79: HR, 0.96, 95%CI: 0.81‐1.14). An increasing number of MetS components was also associated with heightened dementia risk, specifically in late mid‐life (*P* trend = 0.004). Those with a prolonged duration of MetS had a 26% increased risk of developing dementia (HR 1.26, 95%CI: 1.13‐1.40) when compared to those with consistently low MetS. No association was found for increasing MetS (HR, 1.01, 95%CI: 0.88‐1.17).

**Conclusion:**

Late mid‐life MetS was associated with a significantly increased risk of developing dementia. A prolonged duration of living with MetS also elevated dementia risk. These findings provide insights regarding critical periods of dementia risk in the context of MetS, and highlight the importance of not only considering the presence/absence of MetS, but also exposure duration when devising dementia prevention strategies.

